# Can a Multidisciplinary Weight Loss Treatment Improve Motor Performance in Children with Obesity? Results from an Observational Study

**DOI:** 10.3390/healthcare11060899

**Published:** 2023-03-21

**Authors:** Francesca Gallè, Giuliana Valerio, Espedita Muscariello, Ornella Daniele, Valentina Di Mauro, Simone Forte, Teresa Mastantuono, Roberta Ricchiuti, Giorgio Liguori, Pierluigi Pecoraro

**Affiliations:** 1 Department of Movement Sciences and Wellbeing, University of Naples “Parthenope”, Via Medina 40, 80133 Naples, Italy; 2 Nutrition Unit, Department of Prevention, Local Health Authority Napoli 3 Sud, Via Montedoro 47, Torre del Greco, 80059 Naples, Italy

**Keywords:** childhood, body composition, body weight, lifestyle, reaction time, motor performance, physical activity

## Abstract

In the last two decades, the relationship between weight status and children’s motor skill competence has been receiving increasing attention, given its possible role in the prevention and treatment of obesity. This study aimed to evaluate the effect of a multidisciplinary obesity treatment on motor performance in a sample of Italian children and adolescents. Visual and auditory reaction time (VRT and ART), vertical jump elevation (VJE) and power (VJP), body mass index (BMI) and BMI-standard deviation score (BMI-SDS), waist circumference (WC), body composition, dietary habits and physical activity (PA) levels were assessed at baseline and at 6- and 12-month follow-up. Significant improvements were observed in BMI-SDS and FFM, diet and PA levels. Adolescents showed significant improvements in VRT and ART. Jump elevation and power increased in both children and adolescent subgroups. Girls exhibited greater changes than boys in both VRT and ART and VJP but lower changes in VJE. VRT improvement was related to age (OR = 0.285, 95%CI 0.098–0.830, *p* = 0.021) and FFM (OR = 0.255, 95%CI 0.070–0.933, *p* = 0.039). An increase in VJE was associated with BMI-SDS (OR = 0.158, 95%CI 0.036–0.695, *p* = 0.015) and with PA level (OR = 19.102, 95%CI 4.442–82.142, *p* < 0.001); the increase in VJP was related with the increase in PA (OR = 5.564, 95%CI 1.812–17.081, *p* = 0.003). These findings suggest the possible effects of a multidisciplinary obesity treatment on children’s motor competence. Since the improvement in motor skills can increase children’s motivation and adherence to weight loss treatment in the long term, these aspects should be further investigated.

## 1. Introduction

In the last two decades, the growing prevalence of childhood obesity observed in many countries has resulted in many efforts from the scientific community to identify the most effective strategies to prevent or treat this condition [1,2]. Among these, multidisciplinary weight-loss interventions, including personalized nutritional and physical activity (PA) plans and psychological support for children and their families, have been recognized as useful measures for childhood obesity treatment [2,3]. With the aim of increasing adherence to PA recommendations, many studies have been focused on motor competence in obese children, showing that this is inversely related to their weight status [4,5,6]. Indeed, well-developed motor skills are considered a precursor of lifelong engagement in physical activity. Differences in motor performance between children with and without obesity have been found for gross motor tasks, as a mechanical consequence of excess body mass, and even for fine motor tasks, which are predominantly influenced by the ability to process sensory information and transmit proper muscle commands [7,8,9,10].

Carrying out everyday activities requires that children manage gross to fine motor coordination, and at the same time, motor skill competence represents a key determinant of PA engagement [4,11,12,13]. Therefore, the relationship between weight status and children’s motor skill competence is receiving increasing attention, given its possible role in the prevention and treatment of obesity [14,15,16,17]. The literature shows that children with obesity have poorer gross motor coordination performance than their healthy-weight peers, which highlights that weight status affects children’s motor competence levels [9]. Accordingly, weight loss related to obesity treatment interventions may have a beneficial effect on motor skills. In adults and adolescents, weight loss is associated with significant improvements in muscle function, motor control and performance [18,19]. In children, however, research has been so far mainly focused on the effects of weight reduction programs on body fat and other health-related measures, PA levels and physical fitness performance [17]. Studies examining whether weight loss achieved by attending a multidisciplinary program for obesity treatment may also improve children’s motor skill competence and coordination in the long term are still scarce [20,21].

Reaction time (RT) is the time interval between the application of a stimulus and the appearance of a proper voluntary response in a subject [22]. The measurement of RT is considered a simple and valuable cognitive test to evaluate the time needed to initiate and execute a given action [23]. Some factors, such as age and PA levels, have been found to be associated with RT [22,24,25]. Furthermore, some studies have shown a relationship between visual or auditory RT and overweight/obesity, with contradictory results [10,22,24,26,27,28,29].

Therefore, it would be interesting to examine whether perceptual-motor performance can improve in the long term along with weight reduction in children with obesity attending a multidisciplinary obesity treatment intervention.

Since 2018, a second-level outpatient service for childhood obesity treatment based on the multidisciplinary cooperation of nutritionists, psychotherapists and kinesiologists, is active in the province of Naples, a county town of the Campania region, south Italy [30]. The aim of this study was to evaluate the effect of this multidisciplinary intervention on weight-related parameters, visual and auditory RT and jump performance in children participating in the treatment. We hypothesized that children following the program would show improvements in both areas (weight and motor skills) with respect to the baseline measures at the start of treatment. It was expected that the improvement in motor performance would be associated with parameters related to weight loss. Possible gender differences were also explored.

## 2. Materials and Methods

### 2.1. Study Design

This was an observational study performed to assess anthropometric and motor performance changes in a sample of children and adolescents undergoing a multidisciplinary obesity treatment. All the activities were performed respecting the principles of the Declaration of Helsinki. The study was approved by the Local Health Authority “Napoli 3 Sud” of Naples review board (Deliberation n. 92 of 31 January 2020).

### 2.2. Participants and Setting

Children and adolescents with obesity attending the outpatient clinic “Second Level Assistance Center for Diabetes and Obesity in Childhood” of the Local Health Authority “Napoli 3 Sud” were invited to participate in the study.

Parents or guardians of the outpatients who accepted to participate signed informed consent to the use of their data. Participants were consecutively enrolled. All the activities related to the intervention and the data collection were performed in the outpatient clinic by the same professionals.

### 2.3. Intervention

The multidisciplinary intervention has been previously described [30]. Briefly, it is structured in monthly sessions, which include (a) nutritional counseling, with dietary assessment and measure of anthropometric parameters, (b) PA counseling, with an assessment of daily activities and prescription of tailored PA protocols, and (c) motivational support for lifestyle change and maintenance. Each activity is performed by a trained professional in the presence of parents/guardians.

### 2.4. Outcomes

Demographic features of children were collected at the first visit through parents’/guardians’ interviews.

Participants were observed for a twelve-month period from November 2019 to September 2021. The following outcomes were assessed at baseline (indicated as T_0_), after six months (T_6_) and at 1-year follow-up (T_12_).

Anthropometric measurements—body mass index (BMI), waist circumference (WC), bioimpedance analysis and functional tests (Vertical Jump Test-VJT; Reactive tests)—were performed at each visit. Weight and height were measured with a weight scale and altimeter (Wunder Model C201) according to standardized procedures by the same investigator, specifically trained. The BMI was calculated and expressed in kg/m^2^ and converted into BMI standard deviation score (BMI-SDS) [31].

WC was measured through a measuring tape (Seca, Hamburg, Germany) and expressed in cm. Body composition was assessed by bioelectrical impedance analysis (DS Medica, Milan, Italy); fat mass (FM) and fat-free mass (FFM) were expressed both in percentages and kilograms.

Visual reaction time (VRT) and auditory reaction time (ART) were measured by using the OptoJump^TM^ infrared system (Optojump, Microgate, Bolzano, Italy). Each subject was invited to execute a jump inside the measurement area as quickly as she/he could when receiving a visual or auditory stimulus from the monitor. The Optojump^TM^ system recorded the reaction time.

The lower-limb strength was assessed through the squat jump test (SJT). The OptoJump^TM^ infrared system was used to measure the maximum vertical jump with the hands placed aside. Vertical jump elevation (VJE) and power (VJP) were measured by computing the time between contact time and flight time. Three attempts were allowed, with a break of one minute between each attempt; the best performance was recorded for each participant. VRT and ART were expressed in seconds; jump elevation was expressed in centimeters and jump power was expressed in Watt/kg.

Dietary habits were assessed through the 16-item KIDMED questionnaire, a tool widely used to measure the degree of adherence to the Mediterranean diet (MD) model in children and adolescents. The KIDMED total score ranges between 0 and 12 and defines three categories: poor (≤3), average (4–7) and good (≥8) adherence to MD [32].

In order to assess possible changes in PA levels in the course of the treatment, the Physical Activity Questionnaire for Children (PAQ-C) [33] was administered to all the participants at baseline and at both follow-up measurements. The PAQ-C includes 10 items: the first item is a checklist including several common sports, leisure activities and games; items 2–8 aim at assessing activity during the specific day, including physical education class, recess, lunch, immediately after school, evening and the weekend, with two additional questions to assess overall activity patterns during the week. The ninth item regards the frequency of activities performed each day during the week, and item 10th focuses on child health. Each question, except item 10th, is scored using a scale that ranges from 1 to 5. The mean of the items is used to calculate the final PAQ-C summary score.

The test-retest reliability of both KIDMED (κ_w_ = 0.591; 95%CI 0.485, 0.696) and C-PAQ (males, *r* = 0.75 and females, *r* = 0.82) has been measured in previous studies [33,34].

### 2.5. Statistical Analyses

All the variables examined were checked for normal distribution. According to the data distribution, mean and standard deviation (SD) or median and interquartile ranges (IQR) values were used to describe the variables and their comparison between times. The significance of the changes registered throughout the three times was evaluated through ANOVA or Friedman’s test for related samples. Considering the width of the participants’ age range, the comparisons were performed separately for children (7–11 years) and adolescents (12–17 years). The motor outcomes were also compared between baseline and 12-month follow-up in female and male subgroups by using the Student’s t-test for related samples or the Wilcoxon’s test. Multinomial regression analyses were performed to highlight possible relationships between changes in measured outcomes (VRT, ART, jump height and power) and changes in BMI-SDS, FFM and CPAQ category after 12 months of follow-up in the whole sample. To this aim, all these variables were dichotomized as follows. Increase or no change in BMI-SDS, VRT and ART were expressed with a “0” value, and their decrease was expressed as “1”; reduction or no change in FFM, VJE, VJP, KIDMED and CPAQ scores were coded as “0” while their increase was coded as “1”. All the regression analyses were carried out, controlling for age and gender.

## 3. Results

On a total of 248 admitted children and adolescents, 82 youths (45.1% F, mean age 11.1 ± 2.5, range 7–17.3 years) for whom three measurements were available during the follow-up (at the beginning, after 6 and 12 months) were examined.

Table 1 and Table 2 show the changes that occurred in the mean or median values of the examined variables across the three times of follow-ups in the group of children (n = 40) and adolescents (n = 42), respectively, with the corresponding *p*-values.

As for the anthropometric parameters, a significant decrease in BMI-SDS and an increase in FFM (kg) was observed across the three times in both children and adolescents. Children also showed a significant decrease in BMI since baseline.

With regard to the other parameters, the decrease in VRT and ART was significant only in the adolescents’ group and not in children, while jump parameters improved significantly in both groups.

Even the adherence to MD and levels of PA of both children and adolescents significantly increased over the time considered.

**Figure 1:**
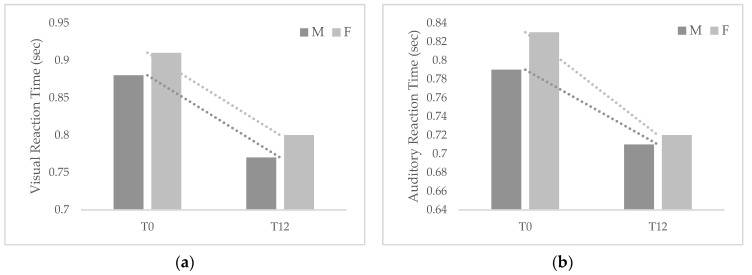
Changes occurred between baseline and 1-year follow-up in visual (**a**) and auditory (**b**) reaction times among male (M) and female (F) participants.

**Figure 2:**
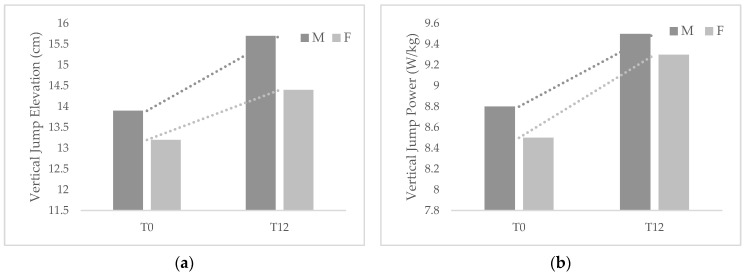
Changes occurred between baseline and 1-year follow-up in jump elevation (**a**) and jump power (**b**) among male (M) and female (F) participants.

Figure 1 and Figure 2 show the changes that occurred in reaction times and jump parameters among participants by gender after 1-year follow-up.

Although both groups showed improvements in the examined parameters, girls exhibited greater changes in both VRT and ART and jump power but lower changes in jump height than boys. VRT changed significantly between T0 and T12 in both males (Δ = −0.12, *p* < 0.01) and females (Δ = −0.11, *p* < 0.01), while ART changed significantly in females (Δ = −0.11, *p* = 0.01) but not in males (Δ = −0.07, *p* = 0.057). VJE changed significantly in males (Δ = 1.79, *p* < 0.01) and females (Δ = 1.19, *p* < 0.01), as well as VJP (males Δ = 0.74, *p* < 0.01; females Δ = 0.85, *p* = 0.01).

As for the regression analyses, VRT improvement was found to be inversely related to age (OR = 0.285, 95%CI 0.098–0.830, *p* = 0.021) and with changes in FFM (OR = 0.255, 95%CI 0.070–0.933, *p* = 0.039); no relationships were found between changes in ART and those detected in the other parameters. An increase in VJE was found to be associated with BMI-SDS (OR = 0.158, 95%CI 0.036–0.695, *p* = 0.015) and with PA level (OR = 19.102, 95%CI 4.442–82.142, *p* < 0.001); even the increase in VJP was positively related with the increase in PA (OR = 5.564, 95%CI 1.812–17.081, *p* = 0.003).

## 4. Discussion

A multidisciplinary intervention in children with obesity has the main objective of reducing excess weight through a permanent change in eating habits and lifestyle while promoting functional mobility and health-related quality of life. In this study, an improvement in diet, physical activity and weight-related outcomes were found in both children and adolescents after the 1-year weight-loss intervention, along with an improvement in reaction times and jump parameters.

As for reaction times, significant improvements were observed in adolescents and in both male and female participants throughout the intervention, suggesting a correlation between weight loss and these outcomes, especially in the higher age class. However, in the regression analyses, only the VRT improvement was found to be related to the increase in fat-free mass. These results are partially in line with those of Moradi et al., who, in 2017, examined the relationship between RT and weight status in a sample of 350 9–12 years-old schoolboys [28]. Among the various RT tasks and obesity indices used, they did not detect significant relationships between ART and BMI, %fat, WC and waist-to-height ratio (WHtR), but just VRT was found to be significantly related with %fat (but not with BMI, WC and WHtR).

The inverse relationship found between visual reaction time and age was previously reported in the literature [22,24,35,36,37]. Therefore, it is possible that the stronger difference observed in reaction time changes among adolescents from our sample is related to their different growth stages. Longer follow-up periods and the comparison with a gender and age-matched control group of children with obesity who do not participate in the multidisciplinary treatment can be useful to clearly define the possible effects of the intervention on children’s reaction time.

The improvement in jump performance, which is a proxy of leg strength, and the association found between jump elevation and BMI-SDS are in accordance with previous literature in this field. The studies by Lazzer et al. showed similar improvements in lower limb power along with body weight and composition improvements even after short obesity treatment interventions [14,15]. The study by D’Hondt et al. reported that the amount of weight loss achieved by children participating in a multidisciplinary residential treatment for overweight/obesity explained 26.9% of the variance in gross motor skills, including jumping performance [21]. On the basis of this finding, the authors concluded that a multidisciplinary residential treatment and concomitant weight loss could be considered an important means to improve gross motor coordination in children with obesity, which in turn may promote their participation in PA. In fact, participation in PA can be easier in children with a high level of motor competence, while children with a lower level of motor competence tend to avoid movement difficulties and may be engaged in sedentary activities [38]. This aspect should be considered in order to increase adherence to the PA plan proposed throughout the treatment. In our study, the improvement observed in jump height and power was found to be related to the increase in PA level. This further underlines the relationship between motor skills and motivation to PA.

This study has some important limitations. First of all, the lack of comparison did not allow us to verify the association between the outcomes and the intervention. Furthermore, it should be considered that the adherence to dietary and PA plans was not objectively monitored but only self-reported. However, it should be noted that both KIDMED and PAQ-C are widely used and have been shown to be adequate for investigating changes in children’s behaviors [38,39]. 

The results of this investigation may contribute to the definition of the best methods to assess the effectiveness of multidisciplinary obesity treatments besides weight loss. The longitudinal design of the investigation and the analysis of children’s body composition and perceptual-motor competence, a field still scarcely explored, represent other strengths of this study.

## 5. Conclusions

The findings of this study showed a significant improvement in motor performance in children undergoing a multidisciplinary obesity treatment, expressed by a decrease in visual and acoustic reaction time and an increase in jump height and power.

The improvement in the visual reaction was associated with the increase in fat-free mass, and the improvement in jump elevation was related to the decrease in BMI-SDS, suggesting a direct effect of the modified weight and body composition on these skills.

Furthermore, the improvement in jump performance was found to be significantly related to the observed increase in physical activity.

These findings underline the role of multidisciplinary obesity treatment in improving youths’ behaviors and related health conditions and also highlight the possible benefits of this type of intervention on youths’ motor competence.

Since improvement in motor skills can increase children’s motivation and their adherence to weight loss treatment in the long term, further controlled studies performed on wider samples and for longer periods are needed to confirm these findings.

## Figures and Tables

**Table 1:** Changes in children’s (n = 40) anthropometric, motor and behavioral variables across the three times of follow-ups.

Variable	T_0_	T_6_	T_12_	*p*-Value
BMI (kg/m^2^) median (IQR)	26.54 (23.77–28.96)	25.34 ^a^ (23.01–28.43)	25.67 ^c^ (24.14–29.24)	0.019
BMI-SDS mean ± SD	2.02 ± 0.39	1.84 ± 0.44 ^a^	1.84 ± 0.51 ^b^	<0.001
WC (cm) median (IQR)	86.5 (82.0–93.9)	86.0 (78.87–92.37)	88.25 (81.75–94.37)	0.064
Phase angle mean ± SD	5.43 ± 0.48	7.60 ± 13.37	5.52 ± 0.82	0.484
FM (%) median (IQR)	35.40 (32.67–39.05)	34.15 ^a^ (31.02–38.70)	35.25 (32.22–39.60)	0.151
FFM (Kg) mean ± SD	32.48 ± 5.63	33.49 ± 5.80	35.30 ± 6.57 ^b,c^	<0.001
VRT (s) median (IQR)	0.92 (0.81–1.09)	0.89 (0.80–1.02)	0.85 (0.74–1.06)	0.535
ART (s) mean ± SD	0.86 ± 0.20	0.83 ± 0.21	0.79 ± 0.17	0.397
VJE (cm) median (IQR)	11.55 (9.94–14.47)	12.37 ^a^ (10.83–15.69)	13.41 ^b^ (10.13–15.88)	0.036
VJP (W/kg) median (IQR)	7.91 (7.21–9.03)	8.31 ^a^ (7.67–9.27)	8.63 ^b^ (7.56–9.36)	0.031
KIDMED score median (IQR)	4 (2.25–6.0)	6 (5.0–7.0)	6 (5.0–7.75) ^b,c^	<0.001
CPAQ score median (IQR)	1.55 (1.32–1.88)	1.85 (1.65–2.12)	2.05 (1.51–2.28) ^b,c^	<0.001

BMI: Body Mass Index; WC: Waist Circumference; FM: Fat Mass; FFM: Fat-Free Mass; VRT: Visual Reaction Time; ART: Auditory Reaction Time; VJE: Vertical Jump Elevation; VJP: Vertical Jump Power; PA: Physical Activity. ^a^ significant difference between T_6_ and T_0_; ^b^ significant difference between T_12_ and T_0_; ^c^ significant difference between T_12_ and T_6._

**Table 2:** Changes in adolescents’ (n = 42) anthropometric, motor and behavioral variables across the three times of follow-ups.

Variable	T_0_	T_6_	T_12_	*p*-Value
BMI (kg/m^2^) median (IQR)	30.20 (28.49–33.19)	30.0 (27.93–32.84)	29.95 (27.74–32.80)	0.060
BMI-SDS mean ± SD	2.27 ± 0.57	2.17 ± 0.58	2.15 ± 0.59 ^c^	0.027
WC (cm) median (IQR)	101.0 (96.87–108.0)	101.0 (95.37–108.0)	100.25 (94.50–108.0)	0.122
Phase angle mean ± SD	5.92 ± 0.71	6.01 ± 0.81	6.10 ± 0.91	0.329
FM (%) median (IQR)	37.80 (34.85–41.10)	36.70 (34.07–39.67)	36.05 (32.07–40.15)	0.059
FFM (Kg) mean ± SD	49.10 ± 9.52	50.08 ± 8.99 ^a^	51.78 ± 9.40 ^b,c^	<0.001
VRT (s) median (IQR)	0.83 (0.72–0.98)	0.79 ^a^ (0.64–0.94)	0.68 ^b^ (0.61–0.83)	<0.001
ART (sec) mean ± SD	0.80 ± 0.30	0.74 ± 0.17	0.68 ± 0.15 ^b^	0.030
VJE (cm) median (IQR)	14.80 (11.62–17.89)	15.68 (12.19–19.03)	15.67 ^b^ (13.81–19.31)	<0.001
VJP (W/kg) median (IQR)	9.11 (8.29–10.16)	9.46 (8.38–10.61)	9.50 ^b^ (9.10–11.37)	<0.001
KIDMED score median (IQR)	4.0 (3.0–6.0)	7.0 (4.0–8.0) ^a^	7.0 (5.0–8.0) ^b^	<0.001
CPAQ score median (IQR)	1.75 (1.29–2.09)	2.01 (1.59–2.50) ^a^	2.15 (2.0–2.56) ^b^	<0.001

BMI: Body Mass Index; WC: Waist Circumference; FM: Fat Mass; FFM: Fat-Free Mass; VRT: Visual Reaction Time; ART: Auditory Reaction Time; VJE: Vertical Jump Elevation; VJP: Vertical Jump Power; PA: Physical Activity. ^a^ significant difference between T_6_ and T_0_; ^b^ significant difference between T_12_ and T_0_; ^c^ significant difference between T_12_ and T_6._

## Data Availability

The data presented in this study are available on request from the corresponding author.
